# Phospho-Tau Bar Code: Analysis of Phosphoisotypes of Tau and Its Application to Tauopathy

**DOI:** 10.3389/fnins.2018.00044

**Published:** 2018-02-06

**Authors:** Taeko Kimura, Govinda Sharma, Koichi Ishiguro, Shin-ichi Hisanaga

**Affiliations:** ^1^Laboratory of Molecular Neuroscience, Department of Biological Sciences, Tokyo Metropolitan University, Hachioji, Japan; ^2^Department of Neurology, Graduate School of Medicine, Juntendo University, Bunkyo, Japan

**Keywords:** phos-tag, tau, phosphorylation, Alzheimer' disease, tauopathy, Cdk5, phospho-tau bar code, GSK3β

## Abstract

Tau is a microtubule-associated protein which regulates the assembly and stability of microtubules in the axons of neurons. Tau is also a major component of neurofibrillary tangles (NFTs), a pathological hallmark in Alzheimer's disease (AD). A characteristic of AD tau is hyperphosphorylation with more than 40 phosphorylation sites. Aggregates of hyperphosphorylated tau are also found in other neurodegenerative diseases which are collectively called tauopathies. Although a large number of studies have been performed on the phosphorylation of AD tau, it is not known if there is disease-specific phosphorylation among tauopathies. This is due to the lack of a proper method for analyzing tau phosphorylation *in vivo*. Most previous phosphorylation studies were conducted using a range of phosphorylation site-specific antibodies. These studies describe relative changes of different phosphorylation sites, however, it is hard to estimate total, absolute and collective changes in phosphorylation. To overcome these problems, we have recently applied the Phos-Tag technique to the analysis of tau phosphorylation *in vitro* and *in vivo*. This method separates tau into many bands during SDS-PAGE depending on its phosphorylation states, creating a bar code appearance. We propose calling this banding pattern of tau the “phospho-tau bar code.” In this review article, we describe what is newly discovered regarding tau phosphorylation through the use of the Phos-Tag. We would like to propose its use for the postmortem diagnosis of tauopathy which is presently done by immunostaining diseased brains with anti-phospho-antibodies. While Phos-tag SDS-PAGE, like other biochemical assays, will lose morphological information, it could provide other types of valuable information such as disease-specific phosphorylation.

## Introduction

Tau is a microtubule (MT)-associated protein (MAP) predominantly expressed in the axons of neurons (Dehmelt and Halpain, 2005; Iqbal et al., 2016; Wang and Mandelkow, 2016). Tau is a phosphoprotein that is targeted by a number of protein kinases. The phosphorylation of tau regulates its functions, namely the assembly and stabilization of MTs (Lindwall and Cole, 1984; Iqbal et al., 2016; Wang and Mandelkow, 2016). Additionally, it is well known that hyperphosphorylated tau is a major component of neurofibrillary tangles (NFTs) in Alzheimer's disease (AD) (Ballatore et al., 2007; Spillantini and Goedert, 2013; Wang et al., 2013; Arendt et al., 2016). There are many other neurodegenerative diseases in which deposits of hyperphosphorylated tau are found. These diseases are collectively called tauopathies (Lee et al., 2001). The fact that Frontotemporal dementia with Parkinsonism linked to Chromosome 17 (FTDP-17), a tauopathy, is caused by the mutation of the tau gene *MAPT* indicates that tau may be a causative factor for other tauopathies in addition to FTDP-17 (Hutton et al., 1998; Poorkaj et al., 1998; Spillantini et al., 1998). It is not known how tau gains neuronal toxicity, however, several possibilities such as oligomerization, aggregation, or hyperphosphorylation have been proposed. It is unclear whether or not the hyperphosphorylation is a cause or result of disease, however, the immunostaining of postmortem brains with anti-phospho-tau antibodies such as AT8, AT180, and PHF1 is usually used as a definitive diagnosis of AD and tauopathy. Therefore, extensive efforts have been made to identify phosphorylation sites and the hyperphosphorylation mechanism. Nevertheless, it is not yet known how this phosphorylation is regulated not only under disease conditions but also in normal brains.

Comprehensive analysis of tau phosphorylation by mass spectroscopy has revealed more than 40 phosphorylation sites in AD pathological tau (Morishima-Kawashima et al., 1995; Hanger et al., 2007). Since antibodies against many of these phosphorylation sites are now commercially available, phosphorylation of tau is currently analyzed using those phosphorylation-site specific antibodies in both physiological and pathological studies. While their use is relatively easy and they are sensitive enough to detect slight changes in phosphorylation levels, there are several unavoidable limitations (described later in detail). When proteins have many phosphorylation sites it is hard to estimate the absolute degree of *in vivo* phosphorylation and discern any combinations of these phosphorylation sites. To answer these difficult but important questions we applied the Phos-Tag SDS-PAGE method to the analysis of tau phosphorylation *in vitro* and *in vivo* (Kimura et al., 2016a,b). We found that tau consists of many bands, resembling a bar code, which indicates heterogeneous phosphorylation in cells and brains. Further, the banding patterns were different depending on phosphorylation states. We call this phosphorylation-dependent banding pattern of tau the “phospho-tau bar code.” We think that the phosphorylation profile would be very useful to identify and diagnose different tauopathies if their phosphorylation is distinctive. Here, we introduce the use of the Phos-tag method in the analysis of tau phosphorylation in physiology and pathology.

## Complicated phosphorylation of tau in cells and brains

**Figure 1:**
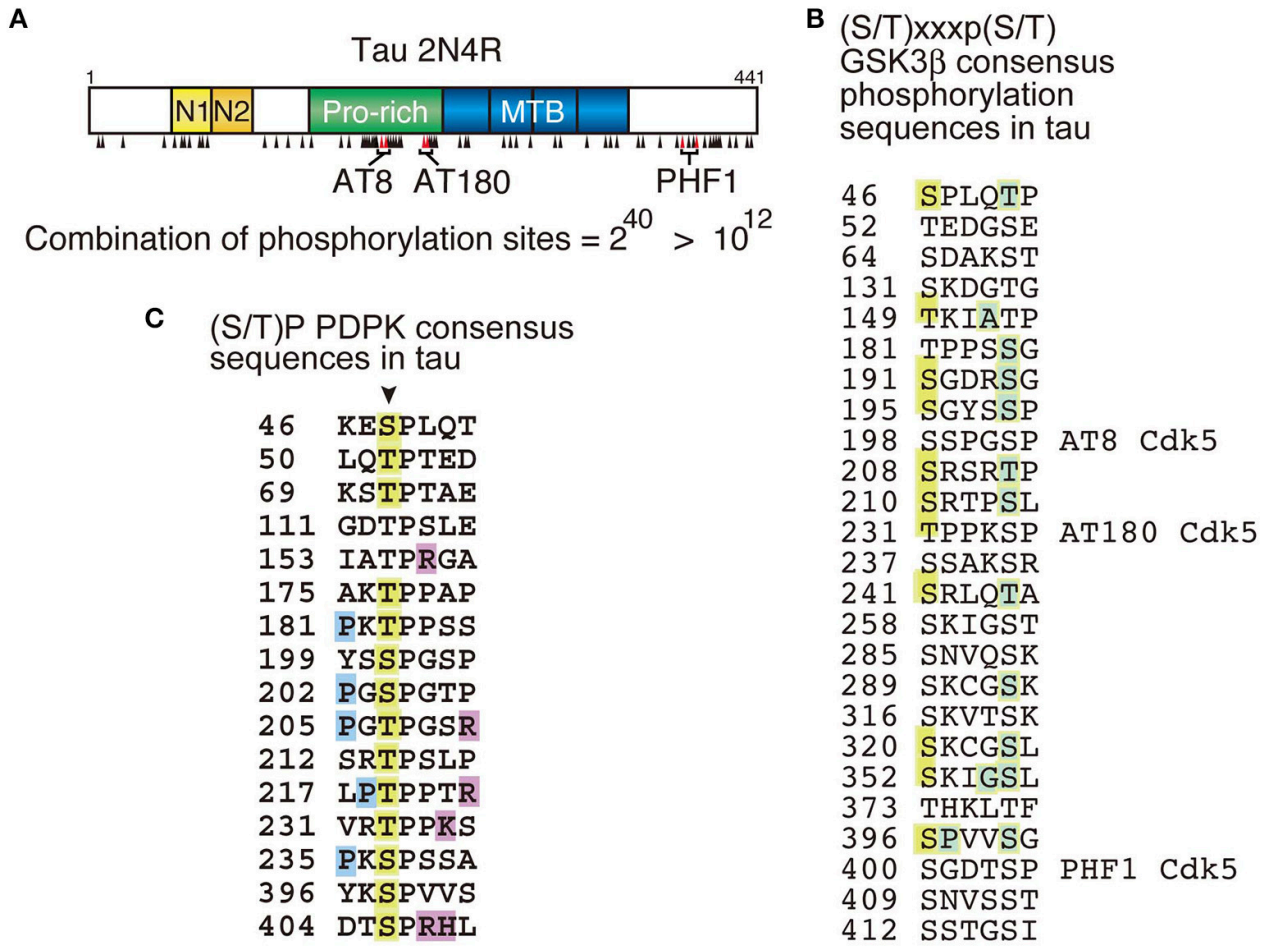
Phosphorylation sites in tau molecule. **(A)** The longest human tau isoform is composed of 441 amino acids with four microtubule-binding (MTB) repeats in the C-terminal half. Phosphorylation sites are indicated by black arrowheads. AT8 (Ser202 and Thr205), AT180 (Thr231 and Ser235) and PHF1 (Ser396 and Ser404) are phosphospecific antibodies frequently used for the postmortem diagnosis of tauopathy and their epitopes are indicated. The number of phosphorylation combination, if all sites are phosphorylated independently, is indicated below. **(B)** Amino acid sequences conforming to the GSK3β consensus sequences, (S/T)xx(x)p(S/T), in tau. There are 25 such sequences and 12 sites are reported to be phosphorylated (orange). The site in the C-terminal sides known to be phosphorylated are indicated by green. **(C)** Ser/Thr-Pro {(S/T)P} sequences in tau targeted by proline-directed protein kinases (PDPK). Arrow indicates Ser or Thr in (S/T)P sequences. Orange is the reported phosphorylation sites, blue is proline (P) conforming to the consensus sequence {Px(S/T)P or P(S/T)P} for MAPK, and magenta is basic amino acids at the C-terminal site which makes Ser or Thr phosphorylation sites favorable for Cdk5.

Tau may be one of the most complicatedly phosphorylated proteins. Tau has 45 serine, 35 threonine and 5 tyrosine residues, resulting in a total of 85 possible phosphorylation sites in the longest human tau isoform composed of 441 amino acids (Goedert et al., 1989). Among them, more than 40 sites are reported to be phosphorylated (Figure 1; Chauhan et al., 2005; Hanger et al., 2007; Wang et al., 2013; Iqbal et al., 2016), and most reside in the Pro-rich region and C-terminal tail region flanking the MT-binding repeats (MTBs). The high density of phosphorylation could be, at least partly, due to an unfolded and extended structure of tau enabling protein kinases to easily access their target sites in consensus phosphorylation sequences. The total number of phosphorylation sites were compiled from data reported in a large number of publications. While it is not likely that a single tau molecule is phosphorylated at all of these sites, it is also unlikely that phosphorylation at all of these sites is completely independent (Hernández et al., 2003). If they were phosphorylated independently, the number of tau phosphorylation combinations would be ~10^12^ (= 2^40^) (Figure 1), an astronomical figure. It is important to understand which sites are phosphorylated in which occasions. A number of phosphorylation sites have been found to regulate MT-binding and -assembly activity of tau and to be involved in development, morphogenesis, and the maintenance of axons in neurons (Lindwall and Cole, 1984; Dehmelt and Halpain, 2005; Wang and Mandelkow, 2016). While those sites are suggested to be in the Pro-rich region and MTB repeats domain, their site-specific functions are not completely understood. Other sites are abnormally phosphorylated in aggregates in AD brains. However, it is not clear which sites contribute to the aggregate formation or are just phosphorylated within the aggregates.

Phosphorylation is the balance of protein kinase and protein phosphatase activity. Several tau kinases such as PKA, CaMKII, PKC, and MAPKs are transiently activated by external or internal stimuli, and their target sites should also be phosphorylated only transiently in healthy neurons and brains. In contrast, several Ser/Thr-Pro {(S/T)P} phosphorylation sites in tau are constitutively phosphorylated which suggests that they are phosphorylated by house-keeping kinase–protein kinases that are active in resting cells (Kimura et al., 2014). However, it is still unclear which sites are phosphorylated, and to what extent and in what context they are phosphorylated. Therefore, the tau phosphorylation is often mentioned as just “phosphorylation” without considering context-dependency.

Tau phosphorylation has several other characteristics that make its phosphorylation complex. A lot of sites are phosphorylated by multiple protein kinases (Chauhan et al., 2005; Hanger et al., 2009; Wang et al., 2013). For example, Ser262 in KXGS motifs in the repeat domain is phosphorylated by MARK, PKA and CaMKII with different activation mechanisms (Drewes et al., 1995; Sironi et al., 1998; Ando et al., 2016). There are 16 (S/T)P sequences in tau, many of which are known as abnormal phosphorylation sites in AD brains (Figure 1C; Kimura et al., 2014). Those (S/T)P sites are targeted by proline-directed protein kinases (PDPKs) such as MAPK (ERK, JNK, and p38 SAPK), GSK3β, Cdk5-p35/p25, and Dyrk1A (Chauhan et al., 2005; Ryoo et al., 2007; Hanger et al., 2009). Since these PDPKs display different substrate preferences; ERK prefers Px(S/T)P or P(S/T)P sequence, Cdk5-p35/p25's consensus is (S/T)Px(K/R/H), and GSK3β phosphorylates (S/T)XXXp(S/T) sequence in addition to (S/T)P sites; their phosphorylation profiles of tau should be different. In fact, some differences are shown by 2D-phospho-peptide mapping–the method which displays the whole phosphorylation profile at once (Illenberger et al., 1998; Sakaue et al., 2005). However, because this method requires isotope labeling of proteins, it is almost impossible to apply to *in vivo* phosphorylation studies. Further with respect to phosphorylation by a single kinase, there are major and minor phosphorylation sites. It has been recently reported that the phosphorylation at Thr205 specifically inhibits amyloid beta (Aβ) toxicity; (Ittner et al., 2016). In that paper, authors examine phosphorylation of tau by p38γ *in vitro* and in cell and in mouse brains using phospho-specific antibodies and Mass spectrometry. They showed four major sites at Ser199, Thr205, Ser396, and Ser404 with 14 minor sites in an *in vitro* kinase assay, but Thr205 alone is strongly phosphorylated in cells and brains overexpressing p38γ. This kind of comprehensive analysis is not always done in every laboratory. More often the phosphorylation sites are discussed without considering quantitative aspects. About 8–11 sites in tau are reported to be Cdk5-p35/p25 phosphorylation sites (Chauhan et al., 2005; Hanger et al., 2009). However, if phosphorylation sites are examined by biochemical methods, it turns out that there are only 3 or 4 major sites located at Ser202 or Thr205, Ser235, and Ser404 (Wada et al., 1998; Sakaue et al., 2005; Kimura et al., 2014). Other sites might be detected with anti-phospho-antibodies which are sensitive enough to detect small levels of phosphorylation, but not distinguish between major and minor phosphorylation sites. Interestingly, these major sites are phosphorylated in consecutive order from the C-terminal site: Ser404, Ser235, and then Thr205 (Kimura et al., 2016b). This kind of information can be determined if the total phosphorylation profile is seen.

The second complicating factor in tau phosphorylation is the ordered phosphorylation, or phosphorylation affected by prior phosphorylation. It is well known that GSK3β is a hierarchical kinase acting on a target site which has prior phosphorylation at +4 (or +3) site, (S/T)xx(x)p(S/T), by a priming kinase. There are 25 such sequences in tau, and 12 sites are indeed phosphorylated, even though not all of them have the primary phosphorylation (Figure 1B). This disagreement between the consensus and real phosphorylation sites may arise from an incomplete analysis with anti-phospho-antibodies. Pathological phosphorylation sites at AT8, AT180 and PHF1 by GSK3β are primed by Cdk5-p35/25 (Li et al., 2006; Kimura et al., 2014). Therefore, tau phosphorylation by GSK3β in cultured cells is dependent on the coexpression of Cdk5-p35. In other words, neurons expressing endogenous Cdk5-p35 or in the case of non-neuronal cells, coexpression with Cdk5-p35 is required for tau phosphorylation by GSK3β. More complicatedly, phosphorylation of tau at the AT-8 (and/or PHF-1) site by GSK3β is primed by PKA phosphorylation at Ser214 even though their phosphorylation sites are separated (Liu et al., 2002, 2004), while GSK3β phosphorylation at Thr212 enhances phosphorylation at Ser214 by PKA (Zheng-Fischhöfer et al., 1998). Phosphorylation by Cdk5 is also influenced, but negatively, by prior phosphorylation. For example, it has been shown that Cdk5-p35/p25 phosphorylates Ser202 and Thr205 at the AT8 site (Shahpasand et al., 2012), but they are not phosphorylated at the same time by Cdk5-p35/p25. In *in vitro* phosphorylation of tau by Cdk5, Cdk5-p35/p25 phosphorylates Thr205 faster than Ser202, and when Thr205 is phosphorylated once, Ser202 phosphorylation by Cdk5 is suppressed (Kimura et al., 2016b). In contrast, in cultured cells where there is endogenous protein kinase(s) phosphorylating Ser202 strongly, Cdk5 can no longer target the Thr205 of tau with Ser202 phosphorylation (Kimura et al., 2016b). However, if tau binds to microtubules, these two sites can be phosphorylated simultaneously by Cdk5 (Wada et al., 1998).

The third complication in tau phosphorylation is problems in the specificity or reactivity of anti-phospho-specific antibodies. Some phospho-specific antibodies are generated against a phosphopeptide with a single phosphorylation at a site of interest. However, several phosphorylation sites in tau are located very close together and, in extreme cases, are right next to each other. The reactivity of the phospho-specific antibody would be affected by any other phosphorylated sites nearby. We recently encountered such a case where the reactivity of anti-pSer202 varied depending of phosphorylation at Thr205 (Kimura et al., 2016b). We therefore found it difficult to assess the *in vivo* phosphorylation of tau at Ser202 properly with that antibody.

## The role of phosphorylation on cellular localization and structure of tau

Tau is mainly localized to axons of neurons in association with microtubules. The axonal tau is detected with Tau-1 antibody which recognizes nonphosphorylation at Ser199 and Ser202 (Binder et al., 1985), and displays a gradient distribution such that dephosphorylated tau is more abundant at the distal region of the axon (Mandell and Banker, 1996). However, it has recently been shown that a small amount of tau is present in dendrites and dendritic spines (Ittner et al., 2010). Dendritic or dendritic spine tau is phosphorylated at AT8 sites, whose phosphorylation is increased by a brief and nontoxic treatment of neurons with glutamate (Kobayashi et al., 2017). Abnormal accumulation of tau in the cell body and dendrites is the result of mislocalization, which is considered an early event in tau pathology. For example, the treatment of neurons with Aβ oligomers induces the transfer of tau to dendrites with enhanced phosphorylation at 12E8 sites (Ser262 and Ser356), which are mediated by MARK, SAD or p70S6K localized in dendrites and spines (Zempel et al., 2010). A fraction (10 ~ 20%) of tau binds to plasma membrane (Brandt et al., 1995; Arrasate et al., 2000; Gauthier-Kemper et al., 2011; Pooler et al., 2012). This tau is not phosphorylated, at least at AD sites such as AT8, PHF-1, and AT-180 (Maas et al., 2000; Pooler et al., 2012). The interaction with membranes is increased by inhibiting casein kinase I or GSK3β or by a phosphorylation-mimicking mutation at the N-terminal residues (Pooler et al., 2012). Tau is also found in the nucleus where it exists mainly in a dephosphorylated state (Loomis et al., 1990; Greenwood and Johnson, 1995; Sultan et al., 2011). Thus, phosphorylation regulates cellular localization of tau but the mechanism is not completely understood.

Phosphorylation also controls the conformation of tau. Tau is a naturally unfolded protein. Nevertheless, tau adopts a “paperclip-like shape” conformation where the N-terminal and C-terminal domains fold to approach the repeat domain (Jeganathan et al., 2006). Phosphorylation at AT8 and PHF sites open the structure to move the N-terminal and C-terminal away respectively (Jeganathan et al., 2008). AT8 site phosphorylation extends the N-terminal region, resulting in the increase of the inter-microtubule distance in neurites (Shahpasand et al., 2012). Phosphorylation at the proline-directed (S/T)P motifs regulates the *trans*-to-*cis* isomerization (Lu et al., 1999). Peptidyl-prolyl cis-trans isomerase Pin1 binds to phosphorylated (S/T)P sequences to convert *cis* to a *trans* conformation, which can be dephosphorylated by protein phosphatase 2A (Zhou et al., 2000: Kimura et al., 2013). In particular, the cis conformation at phosphorylated Thr231 has a toxic activity, leading to tau-dependent neurodegeneration (Kondo et al., 2015). AD tau is highly phosphorylated and aggregated (Ballatore et al., 2007; Spillantini and Goedert, 2013; Wang et al., 2013; Arendt et al., 2016). A role for the HSP90-CHIP complex is suggested in pathology of tauopathies via its recognition and selective degradation of phosphorylated tau (Dickey et al., 2007). Tyr phosphorylation of tau also correlates with the formation of tau aggregates (Vega et al., 2005).

## Phos-tag SDS-PAGE; a powerful technique for analysis of overall, quantitative and combinatory phosphorylation

Phosphorylation often reduces the electrophoretic mobility of proteins in Laemmli's SDS-PAGE. These shifts have been considered as evidence of phosphorylation. Neurofilament proteins NF-M and NF-H are typical examples of phosphorylated, shifted proteins. NF-H which is phosphorylated at the KSP sequences in the C-terminal tail region–as many as ~50 sites in the axons of neurons–moves to a position of 200 kDa on Laemmli's SDS-PAGE (Julien and Mushynski, 1998; Pant et al., 2000). Dephosphorylation of NF-H decreases its apparent molecular size to 140 kDa (Julien and Mushynski, 1982; Hisanaga and Hirokawa, 1989). While the up-shift and down-shift can be used as an indication of phosphorylation and dephosphorylation, they do not, however, provide further information on phosphorylation. Moreover, a shift does not always occur in every protein. Larger numbers of proteins, particularly those with a few phosphorylation sites, have an overall unaffected electrophoretic mobility upon phosphorylation.

**Figure 2:**
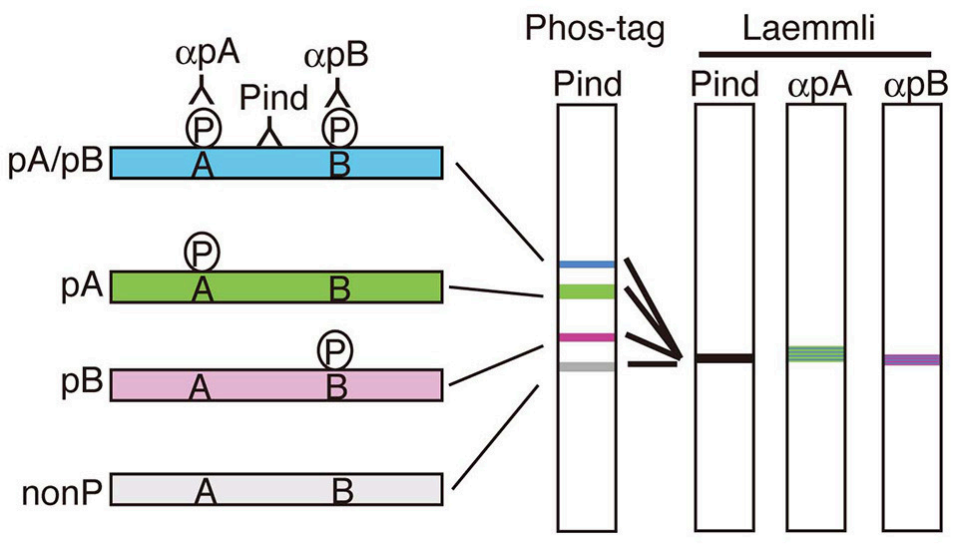
Separation of phosphoisotypes of a protein on Phos-tag SDS-PAGE. If there are two phosphorylation sites, A and B, in a protein, there would be 4 phosphoisotypes, doubly phosphorylated (blue), two single site-phosphorylated (green and red) and nonphosphorylated (left). These four phosphoisotypes can be separated into four bands on Phos-tag SDS-PAGE by shifting upward differently depending on the number and site of phosphorylation (Phos-tag). Phospho-specific antibodies, αpA and αpB, are required to detect the respective phosphorylation in Laemmli's SDS-PAGE, in contrast, the separation in Phos-tag indicates not only phosphorylation but also the degree of phosphorylation detected with a phosphorylation-independent antibody alone (Pind).

Phos-tag SDS-PAGE is a phosphoaffinity electrophoresis method which was developed by Kinoshita et al. (2006). The procedure of Phos-tag SDS-PAGE is simple, similar to that of Laemmli's SDS-PAGE except for the use of Phos-tag acrylamide (commercially available from Wako Chemical) as a separating gel. The Phos-tag is a chemical structure capable of capturing the phosphate on proteins. Therefore, migration of phosphorylated proteins are extraordinarily delayed during electrophoresis due to their binding to the Phos-tag moiety conjugated to acrylamide. Importantly, the degree of the delay, that is the upward shift, is dependent on the number and site of phosphorylation so that phosphorylated proteins are separated into distinct bands depending on their phosphorylation states (Figure 2). Species of proteins with different combinations of phosphorylated sites are designated as “phosphoisotypes” (Kinoshita et al., 2009; Hosokawa et al., 2010). After blotting on a nitrocellulose or PVDF membrane, all phosphoisotyes would be detected with a phosphorylation-independent antibody of a protein of interest (see Kinoshita et al., 2009 more in detail).

There are the following advantages in the use of the Phos-tag method. (1) The total phosphorylation profile of the particular protein is seen at a glance (see Figure 4 for an example). This is because all phosphoisotypes of the protein including the nonphosphorylated one, can be detected on a single blot membrane with a single antibody. It is easy to know whether your protein of interest is phosphorylated *in vivo* or not without prior labeling of proteins or the use of phospho-specific antibodies. The only thing to be cautious of is dephosphorylation and the proteolytic cleavage of proteins during sample preparation. (2) If it is known that the protein is phosphorylated, then a phosphorylation site(s) can be readily determined by the loss of the upward shift with a nonphosphorylation Ala mutant at candidate sites. We have newly identified the phosphorylation sites of several proteins, ataxin 2, drebrin and GRAB, by applying this method (Asada et al., 2014; Tanabe et al., 2014; Furusawa et al., 2017). (3) Once you have determined the phosphorylation states of each band, you may determine the combination of phosphorylated sites even *in vivo* by comparing with the standard profile of phosphoisotypes. (4) The relative ratio of each phosphoisotype can be measured as a percent ratio of the total by densitometric scanning of the blots. We have determined the ratio of phosphoisotypes of p35 Cdk5 activator *in vivo* and found it changes with brain development (Hosokawa et al., 2010; Krishnankutty et al., 2017). It was difficult to obtain this kind of information through the use of previous methods. We think this method would be useful to reveal the age-dependent changes of tau phosphorylation.

Another favorable feature of this technique is its easy and simple implementation. Every laboratory, where immunoblotting is routinely carried out, can employ it immediately without any extra equipment. Further, the method does not require the preparation of phosphospecific antibodies, although it is preferable to use them if possible. Moreover, the samples already prepared for Laemmli's SDS-PAGE can be used for Phos-tag SDS-PAGE as well. In contrast, a weakness of Phos-tag SDS-PAGE is the analysis of phosphoproteins composed of multiple isoforms with different molecular weights such as tau. Because the method depends on the upward mobility shift of phosphorylated isoforms, it would be difficult to distinguish the phosphorylated bands from the larger isoforms. Further, when the number of phosphorylation sites is increased, the banding pattern would become more complicated. This method may be most powerful on single isoform proteins with 2~3 phosphorylation sites such as a p35 Cdk5 activator or GSK3β we have reported (Hosokawa et al., 2010; Krishnankutty et al., 2017). Therefore, it is somewhat challenging to analyze tau by this method. So far we have employed one-dimensional Phos-tag SDS-PAGE, where we have identified most of the phosphorylation sites of a single tau isoform expressed in cultured cells and not *in vivo* tau in mouse brains. However, by increasing resolution, for example, using 2-dimentional SDS-PAGE composed of Laemmli's and Phos-tag, it would become possible to analyze each isoform separately. Otherwise, by developing the isolation method of a single isoform, for example, by immunoprecipitation, we may determine the isoform-specific phosphorylation sites.

## Phospho-tau bar code

**Figure 3:**
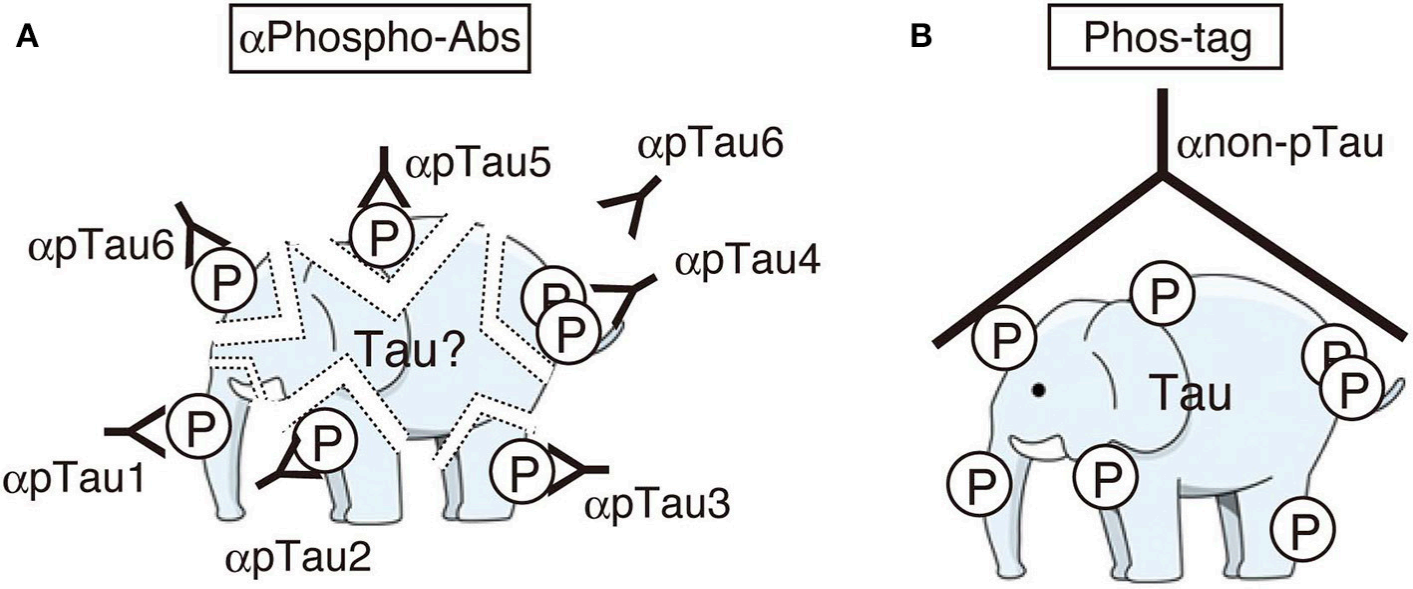
Cartoons representing the images of immunoblotting with many phospho-specific antibodies **(A)** and a phosphorylation-independent single antibody after Phos-tag SDS-PAGE **(B)**. Phosphospecific antibodies detect only part of phosphorylation at each site and some sites can be masked by another phosphorylated site nearby (here the site for αpTau6 is masked by a phosphorylated site which reacts with αpTau4). Phos-tag shows the whole phosphorylation profile at once. An illustration of an elephant was downloaded from the free illustration site at: http://illpop.com/png_animalhtm/elephant_a04.htm.

**Figure 4:**
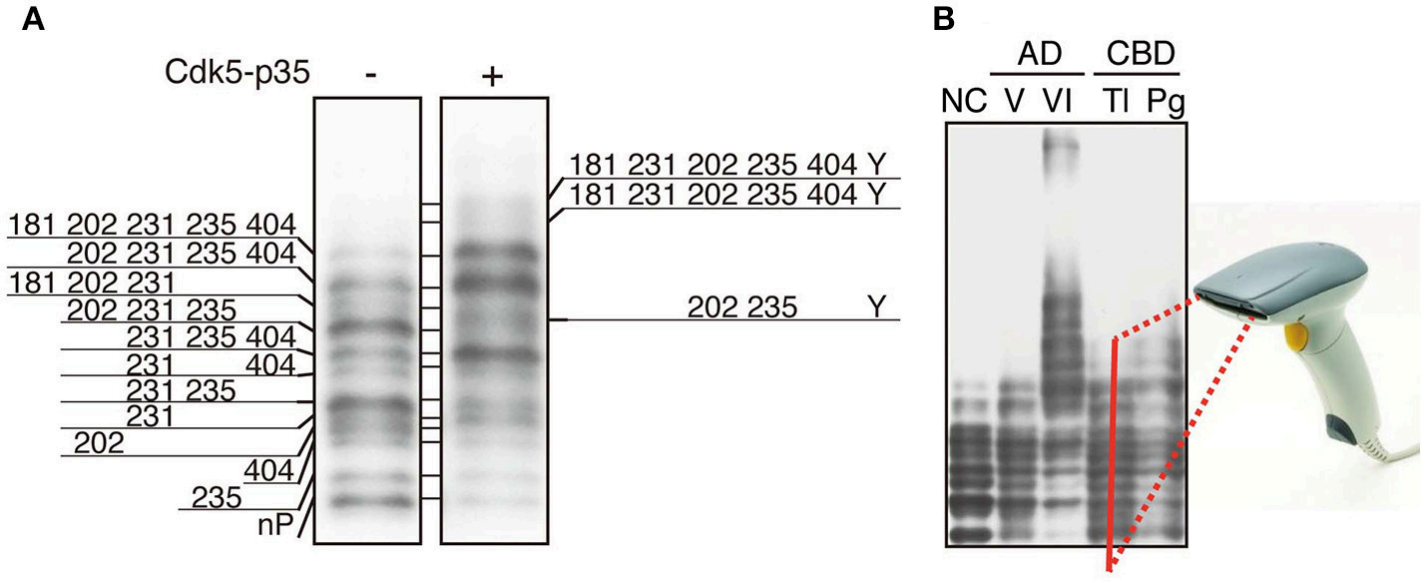
Phosphoisotypes of tau on Phos-tag SDS-PAGE. **(A)** Immunoblotting of tau expressed in COS-7 cells with tau5 in the presence (+) or absence (−) of Cdk5-p35 after Phos-tag-SDS-PAGE, and the phosphorylation sites in each band are indicated by the amino acid number according to the human longest isoform of tau. Y is an unknown phosphorylation site. **(B)** Immunoblotting of tau in human brains after Phos-tag SDS-PAGE. NC, normal control; AD V and VI, Braak stage V and VI of Alzheimer's disease (AD); CBD Tl and Pg, temporal lobe (Tl) and prefrontal gyrus (Pg) of corticobasal degeneration (CBD). We propose that the banding pattern created using Phos-tag SDS-PAGE could be read by a bar code reader and result in a simple method for diagnosing tauopathy. **(A,B)** are reproduced and modified from figures reported previously by Kimura et al. (2016a,b), respectively.

While the identification of tau phosphorylation was initially determined comprehensively by the methods of amino acid sequence and Mass analysis (Watanabe et al., 1993; Morishima-Kawashima et al., 1995), recently most studies use anti-phospho-antibodies to detect and confirm them. The use of anti-phospho-antibodies describe phosphorylation states of corresponding epitope sites, but as described above, however, the heterogeneous and complicated phosphorylation of tau make the interpretation of total and quantitative phosphorylation difficult. It is as difficult to conceive of the total complete picture of tau phosphorylation as a whole, as it is for the blind men touching just part of an elephant to describe the whole animal (Figure 3A). It is desirable to ascertain the total phosphorylation of a protein easily and simply (Figure 3B). In order to reveal the overall phosphorylation profiles of tau, we have applied the Phos-tag method to tau phosphorylation *in vitro*, in cells, and in mouse and human brains (Kimura et al., 2016a,b). In *in vitro* experiments using Cdk5-p25 as a protein kinase, we found that tau was phosphorylated mainly at Thr205, Ser235, and Ser404 sequentially from the C-terminal to N-terminal sites. When expressed in COS-7 cells, tau was separated into as many as 12 bands using Phos-tag SDS-PAGE. Their respective phosphorylation sites were determined and it was found that tau was phosphorylated at 0–5 sites in different combinations (Figure 4A; Kimura et al., 2016b). Coexpression of Cdk5-p35 shifted up each tau bands by phosphorylating Ser404. Through quantification, it was determined that Thr231 was easily phosphorylated in about 50% of total tau molecules expressed in COS-7 cells. Tau in mouse brains was also composed of a number of different phosphoisotypes and this pattern changed with brain development (Kimura et al., 2016a). To us the banding patterns resemble a bar code, thus we call it “Phospho-tau bar code.”

## Applying the phospho-tau bar code to pathological phosphorylation in tauopathy brains

Tau is highly phosphorylated in aggregates in AD brains and possibly in other tauopathy brains. In fact, postmortem diagnosis of tauopathies, including AD, is done by immunohistochemical staining of patients' brains with anti-tau phospho-specific antibodies such as AT8 (Ser202 and Thr205), AT180 (Thr231 and Ser235) and PHF1 (Ser396 and Ser404) (Figure 1A). Since each tauopathy differs in the affected regions and aggregated structures (Ballatore et al., 2007; Spillantini and Goedert, 2013; Wang et al., 2013; Arendt et al., 2016), the immunohistochemistry involving morphological information can serve as diagnosis. In contrast, although it is known by the immunoblotting that isoforms forming aggregates are different depending on tauopathies; both 3R and 4R in AD, 4R in CBD and PSP, and 3R in Pick's disease, it is not used for diagnosis because the procedure is relatively burdensome and the isoform differences may not be sufficient for distinguishing between many tauopathies. However, it has recently been proposed that there are tau strains with distinct structural conformations which could underlie the heterogeneity of tauopahites (Sanders et al., 2014). While the strain specific for each tauopathy has not been established yet, there is a possibility that respective strains might differ in their phosphorylation. We have compared phosphorylation profiles of tau in AD and corticobasal degeneration (CBD) with that of normal control using Phs-tag SDS-PAGE (Kimura et al., 2016a). The expression of 6 tau isoforms with slightly different molecular sizes in human adult brains made analysis extremely difficult, although the use of 3R (tau isoforms with three MTB) or 4R (tau isoforms with four MTB) specific antibodies or phospho-specific antibodies reduced the number of bands to some extent. Even though tau in Sarkosyl-insoluble aggregates prepared from AD patient brains displayed a smeared banding pattern on the immunoblots after Laemmli's SDS-PAGE, their separation on Phos-tag suggests that several phosphorylated tau bands show similar mobility to hyperphosphorylated tau species in brains of perinatal mice or hypothermia mice. This suggests some similarity in phosphorylation between AD and embryonic/perinatal brains, as was previously reported (Morishima-Kawashima et al., 1995). When tau in the brain extracts was analyzed by Phos-tag, tau at Braak stage V of AD contained slightly shifted up bands compared to normal control (Figure 4B), and tau in brains of corticobasal degeneration (CBD) patients displayed a slightly different banding pattern. This different banding patterns of tau between AD and CBD may suggest different phosphorylation states among tauopathies. If distinct phosphorylation profiles will be found among tauopathies, the method can be applied to their diagnosis. Even if this method alone is not enough to make the diagnosis, the obtained information would assist the diagnosis by immunohistochemistry and also be useful for better understanding tau pathology. Further, if the sensitivity of detection will be increased, this method may be applied to diagnosis using cerebrospinal fluids. Moreover, phosphorylation profiles would be used for the development of therapeutic drugs. Several protein kinase inhibitors are in Phase II trials for AD in 2017 (Cummings et al., 2017). Despite considerable efforts, however, success so far is insufficient. If the involvement of a specific kinase in particular processes of different tauopathies is clarified, the more specific strategy could become possible. In addition to the development of small chemical molecules, it would be possible to develop the drug based on the activation or inhibition mechanism of respective protein kinases. One such kinase inhibitor is TFP5, the peptide derived from the amino acid sequence of p35 Cdk5 activator, which inhibits hyperactive Cdk5-p25 specifically *in vivo* (Shukla et al., 2017).

## Conclusion

Tauopathy neurodegenerative disorders including AD are characterized by intracellular inclusions composed of hyperphosphorylated tau. Immunocytochemical detection of tau inclusions with phospho-specific tau antibodies is usually used as a postmortem diagnosis of diseases. The different staining patterns depending on disease type suggest disease-specific phosphorylation but it is not yet clear. It is desirable to use a method which examines the total phosphorylation profiles of tau simply. We recently applied the Phos-tag technique to analyze tau phosphorylation in mouse and human brains, and found that tau is separated into many bands, resembling a bar code of many different phosphoisotypes. Here, we name such a banding pattern of tau on Phos-tag SDS-PAGE the “phospho-tau bar code” and propose its use for the diagnosis of tauopathies.

## Author contributions

TK and SH wrote the whole manuscript. GS and KI made figures and edited the manuscript.

### Conflict of interest statement

The authors declare that the research was conducted in the absence of any commercial or financial relationships that could be construed as a potential conflict of interest.

## Abbreviations

3R: 3 repeats
4R: 4 repeats
AD: Alzheimer's disease
CBD: corticobasal degeneration
Cdk5: cyclin-dependent kinase 5
GSK3β: glycogen synthase kinase 3beta
MAP: microtubule-associated protein
MT: microtubule
MTB: microtubule-binding repeats
MAPK: mitogen-activated kinase
NFT: neurofibrillary tangle
PDPK: proline-directed protein kinase
FTDP-17: frontotemporal dementia and parkinsonism linked to chromosome 17
NF-H: Neurofilament heavy chain
PKA: protein kinase A
PKC: protein kinase C
CaMKII: calcium-calmodulin-dependent protein kinase II
SDS-PAGE: sodium dodecyl sulfate polyacrylamide gel electrophoresis.

## References

[B1] Ando K. Maruko-Otake A. Ohtake Y. Hayashishita M. Sekiya M. Iijima K. M. (2016). Stabilization of Microtubule-Unbound Tau via Tau Phosphorylation at Ser262/356 by Par-1/MARK Contributes to Augmentation of AD-Related Phosphorylation and Aβ42-Induced Tau Toxicity. PLoS Genet. 2:e1005917 10.1371/journal.pgen.1005917 PMC4811436 27023670

[B2] Arendt T. Stieler J. T. Holzer M. (2016). Tau and tauopathies. Brain Res. Bull. 126, 238–292. 10.1016/j.brainresbull.2016.08.018 27615390

[B3] Arrasate M. Pérez M. Avila J. (2000). Tau dephosphorylation at tau-1 site correlates with its association to cell membrane. Neurochem. Res. 25, 43–50. 10.1023/A:1007583214722 10685603

[B4] Asada A. Yamazaki R. Kino Y. Saito T. Kimura T. Miyake M.. (2014). Cyclin-dependent kinase 5 phosphorylates and induces the degradation of ataxin-2. Neurosci. Lett. 563, 112–117. 10.1016/j.neulet.2014.01.046 24486837

[B5] Ballatore C. Lee V. M. Trojanowski J. Q. (2007). Tau-mediated neurodegeneration in Alzheimer's disease and related disorders. Nat. Rev. Neurosci. 8, 663–672. 10.1038/nrn2194 17684513

[B6] Binder L. I. Frankfurter A. Rebhun L. I. (1985). The distribution of tau in the mammalian central nervous system. J. Cell Biol. 101, 1371–1378. 10.1083/jcb.101.4.1371 3930508 PMC2113928

[B7] Brandt R. Léger J. Lee G. (1995). Interaction of tau with the neural plasma membrane mediated by tau's amino-terminal projection domain. J. Cell Biol. 131, 1327–1340. 10.1083/jcb.131.5.1327 8522593 PMC2120645

[B8] Chauhan N. B. Siegel G. J. Feinstein D. L. (2005). Propentofylline attenuates tau hyperphosphorylation in Alzheimer's Swedish mutant model Tg2576. Neuropharmacology 48, 93–104. 10.1016/j.neuropharm.2004.09.014 15617731

[B9] Cummings J. Lee G. Mortsdorf T. Ritter A. Zhong K. (2017). Alzheimer's disease drug development pipeline: 2017. Alzheimers Dement (N. Y) 3, 367–384. 10.1016/j.trci.2017.05.002 29067343 PMC5651419

[B10] Dehmelt L. Halpain S. (2005). The MAP2/Tau family of microtubule-associated proteins. Genome Biol. 6:204. 10.1186/gb-2004-6-1-204 15642108 PMC549057

[B11] Dickey C. A. Kamal A. Lundgren K. Klosak N. Bailey R. M. Dunmore J.. (2007). The high-affinity HSP90-CHIP complex recognizes and selectively degrades phosphorylated tau client proteins. J. Clin. Invest. 117, 648–658. 10.1172/JCI29715 17304350 PMC1794119

[B12] Drewes G. Trinczek B. Illenberger S. Biernat J. Schmitt-Ulms G. Meyer H. E.. (1995). Microtubule-associated protein/microtubule affinity-regulating kinase (p110mark) a novel protein kinase that regulates tau-microtubule interactions and dynamic instability by phosphorylation at the Alzheimer-specific site serine 262. J. Biol. Chem. 270, 7679–7688. 10.1074/jbc.270.13.7679 7706316

[B13] Furusawa K. Asada A. Saito T. Fukuda M. Hisanaga S. (2017). Cdk5 regulates Rab8-dependent axonal outgrowth via phosphorylation of Rab8 guanine-exchange factor Grab. J. Neurosci. 37, 790–806. 10.1523/JNEUROSCI.2197-16.2016 28123016 PMC6597024

[B14] Gauthier-Kemper A. Weissmann C. Golovyashkina N. Sebö-Lemke Z. Drewes G. Gerke V.. (2011). The frontotemporal dementia mutation R406W blocks tau's interaction with the membrane in an annexin A2–dependent manner. J. Cell Biol. 192, 647–661. 10.1083/jcb.201007161 21339331 PMC3044115

[B15] Goedert M. Spillantini M. G. Jakes R. Rutherford D. Crowther R. A. (1989). Multiple isoforms of human microtubule-associated protein tau: sequences and localization in neurofibrillary tangles of Alzheimer's disease. Neuron 3, 519–526. 10.1016/0896-6273(89)90210-9 2484340

[B16] Greenwood J. A. Johnson G. V. (1995). Localization and in situ phosphorylation state of nuclear tau. Exp. Cell Res. 220, 332–337. 10.1006/excr.1995.1323 7556441

[B17] Hanger D. P. Anderton B. H. Noble W. (2009). Tau phosphorylation: the therapeutic challenge for neurodegenerative disease. Trends Mol. Med. 15, 112–119. 10.1016/j.molmed.2009.01.003 19246243

[B18] Hanger D. P. Byers H. L. Wray S. Leung K. Y. Saxton M. J. Seereeram A.. (2007). Novel phosphorylation sites in tau from Alzheimer brain support a role for casein kinase 1 in disease pathogenesis. J. Biol. Chem. 282, 23645–23654. 10.1074/jbc.M703269200 17562708

[B19] Hernández F. Lucas J. J. Cuadros R. Avila J. (2003). GSK-3 dependent phosphoepitopes recognized by PHF-1 and AT-8 antibodies are present in different tau isoforms. Neurobiol. Aging 24, 1087–1094. 14643380 10.1016/j.neurobiolaging.2003.04.002

[B20] Hisanaga S. Hirokawa N. (1989). The effects of dephosphorylation on the structure of the projections of neurofilament. J. Neurosci. 9, 959–966. 2538587 10.1523/JNEUROSCI.09-03-00959.1989 PMC6569983

[B21] Hosokawa T. Saito T. Asada A. Fukunaga K. Hisanaga S. (2010). Quantitative measurement of *in vivo* phosphorylation states of Cdk5 activator p35 by Phos-tag SDS-PAGE. Mol. Cell Proteom 9, 1133–1143. 10.1074/mcp.M900578-MCP200 20097924 PMC2877975

[B22] Hutton M. Lendon C. L. Rizzu P. Baker M. Froelich S. Houlden H.. (1998). Association of missense and 5'-splice-site mutations in tau with the inherited dementia FTDP-17. Nature 393, 702–705. 10.1038/31508 9641683

[B23] Illenberger S. Zheng-Fischhöfer Q. Preuss U. Stamer K. Baumann K. Trinczek B.. (1998). The endogenous and cell cycle-dependent phosphorylation of tau protein in living cells: implications for Alzheimer's disease. Mol. Biol. Cell 9, 1495–1512. 10.1091/mbc.9.6.1495 9614189 PMC25374

[B24] Iqbal K. Liu F. Gong C. X. (2016). Tau and neurodegenerative disease: the story so far. Nat. Rev. Neurol. 12, 15–27. 10.1038/nrneurol.2015.225 26635213

[B25] Ittner A. Chua S. W. Bertz J. Volkerling A. van der Hoven J. Gladbach A.. (2016). Site-specific phosphorylation of tau inhibits amyloid-β toxicity in Alzheimer's mice. Science 354, 904–908. 10.1126/science.aah6205 27856911

[B26] Ittner L. M. Ke Y. D. Delerue F. Bi M. Gladbach A. van Eersel J.. (2010). Dendritic function of tau mediates amyloid-β toxicity in Alzheimer's disease mouse models. Cell 142, 387–397. 10.1016/j.cell.2010.06.036 20655099

[B27] Jeganathan S. Hascher A. Chinnathambi S. Biernat J. Mandelkow E. M. Mandelkow E. (2008). Proline-directed pseudo-phosphorylation at AT8 and PHF1 epitopes induces a compaction of the paperclip folding of Tau and generates a pathological (MC-1) conformation. J. Biol. Chem. 283, 32066–32076. 10.1074/jbc.M805300200 18725412

[B28] Jeganathan S. von Bergen M. Brutlach H. Steinhoff H. J. Mandelkow E. (2006). Global hairpin folding of tau in solution. Biochemistry 45, 2283–2293. 10.1021/bi0521543 16475817

[B29] Julien J. P. Mushynski W. E. (1982). Multiple phosphorylation sites in mammalian neurofilament polypeptides. J. Biol. Chem. 257, 10467–10470. 7202005

[B30] Julien J. P. Mushynski W. E. (1998). Neurofilaments in health and disease. Prog. Nucleic Acid Res. Mol. Biol. 61, 1–23. 10.1016/S0079-6603(08)60823-5 9752717

[B31] Kimura T. Hatsuta H. Masuda-Suzukake M. Hosokawa M. Ishiguro K. Akiyama H.. (2016a). The abundance of nonphosphorylated tau in mouse and human tauopathy brains revealed by the use of phos-tag method. Am. J. Pathol. 186, 398–409. 10.1016/j.ajpath.2015.10.009 26687814

[B32] Kimura T. Hosokawa T. Taoka M. Tsutsumi K. Ando K. Ishiguro K.. (2016b). Quantitative and combinatory determination of in situ phosphorylation of tau and its FTDP-17 mutants. Sci. Rep. 6:33479. 10.1038/srep33479 27641626 PMC5027580

[B33] Kimura T. Ishiguro K. Hisanaga S. (2014). Physiological and pathological phosphorylation of tau by Cdk5. Front. Mol. Neurosci. 7:65. 10.3389/fnmol.2014.00065 25076872 PMC4097945

[B34] Kimura T. Tsutsumi K. Taoka M. Saito T. Masuda-Suzukake M. Ishiguro K.. (2013). Isomerase Pin1 stimulates dephosphorylation of tau protein at cyclin-dependent kinase (Cdk5)-dependent Alzheimer phosphorylation sites. J. Biol. Chem. 288, 7968–7977. 10.1074/jbc.M112.433326 23362255 PMC3597833

[B35] Kinoshita E. Kinoshita-Kikuta E. Koike T. (2009). Separation and detection of large phosphoproteins using Phos-tag SDS-PAGE. Nat. Protocol. 4:1513. 10.1038/nprot.2009.154 19798084

[B36] Kinoshita E. Kinoshita-Kikuta E. Takiyama K. Koike T. (2006). Phosphate-binding tag, a new tool to visualize phosphorylated proteins. Mol. Cell Proteomics 5, 749–757. 10.1074/mcp.T500024-MCP200 16340016

[B37] Kobayashi S. Tanaka T. Soeda Y. Almeida O. F. X. Takashima A. (2017). Local somatodendritic translation and hyperphosphorylation of tau protein triggered by AMPA and NMDA receptor stimulation. EBioMedicine 20, 120–126. 10.1016/j.ebiom.2017.05.012 28566250 PMC5478209

[B38] Kondo A. Shahpasand K. Mannix R. Qiu J. Moncaster J. Chen C. H.. (2015). Antibody against early driver of neurodegeneration cis P-tau blocks brain injury and tauopathy. Nature 523, 431–436. 10.1038/nature14658 26176913 PMC4718588

[B39] Krishnankutty A. Kimura T. Saito T. Aoyagi K. Asada A. Takahashi S. I.. (2017). *In vivo* regulation of glycogen synthase kinase 3β activity in neurons and brains. Sci. Rep. 7:8602. 10.1038/s41598-017-09239-5 28819213 PMC5561119

[B40] Lee V. M. Goedert M. Trojanowski J. Q. (2001). Neurodegenerative tauopathies. Ann. Rev. Neurosci. 24, 1121–1159. 10.1146/annurev.neuro.24.1.1121 11520930

[B41] Li T. Hawkes C. Qureshi H. Y. Kar S. Paudel H. K. (2006). Cyclin-dependent protein kinase 5 primes microtubule-associated protein tau site-specifically for glycogen synthase kinase 3β. Biochemistry 45, 3134–3145. 10.1021/bi051635j 16519508

[B42] Lindwall G. Cole R. D. (1984). Phosphorylation affects the ability of tau protein to promote microtubule assembly. J. Biol. Chem. 259, 5301–5305. 6425287

[B43] Liu F. Iqbal K. Grundke-Iqbal I. Gong C. X. (2002). Involvement of aberrant glycosylation in phosphorylation of tau by cdk5 and GSK-3β. FEBS Lett. 530, 209–214. 10.1016/S0014-5793(02)03487-7 12387894

[B44] Liu S. J. Zhang J. Y. Li H. L. Fang Z. Y. Wang Q. Deng H. M.. (2004). Tau becomes a more favorable substrate for GSK-3 when it is prephosphorylated by PKA in rat brain. J. Biol. Chem. 279, 50078–50088. 10.1074/jbc.M406109200 15375165

[B45] Loomis P. A. Howard T. H. Castleberry R. P. Binder L. I. (1990). Identification of nuclear tau isoforms in human neuroblastoma cells. Proc. Natl. Acad. Sci. U.S.A. 87, 8422–8426. 10.1073/pnas.87.21.8422 1700432 PMC54968

[B46] Lu P.-J. Wulf G. Zhou X. Z. Davies P. Lu K. P. (1999). The prolyl isomerase Pin1 restores the function of Alzheimer-associated phosphorylated tau protein. Nature 399, 784–788. 10.1038/21650 10391244

[B47] Maas T. Eidenmüller J. Brandt R. (2000). Interaction of tau with the neural membrane cortex is regulated by phosphorylation at sites that are modified in paired helical filaments. J. Biol. Chem. 275, 15733–15740. 10.1074/jbc.M000389200 10747907

[B48] Mandell J. W. Banker G. A. (1996). A spatial gradient of tau protein phosphorylation in nascent axons. J. Neurosci. 16, 5727–5740. 8795628 10.1523/JNEUROSCI.16-18-05727.1996 PMC6578967

[B49] Morishima-Kawashima M. Hasegawa M. Takio K. Suzuki M. Yoshida H. Titani K.. (1995). Proline-directed and non-proline-directed phosphorylation of PHF-tau. J. Biol. Chem. 270, 823–829. 10.1074/jbc.270.2.823 7822317

[B50] Pant H. C. Veeranna Grant P. (2000). Regulation of axonal neurofilament phosphorylation. Curr. Top. Cell. Regul. 36, 133–150. 10.1016/S0070-2137(01)80006-6 10842750

[B51] Pooler A. M. Usardi A. Evans C. J. Philpott K. L. Noble W. Hanger D. P. (2012). Dynamic association of tau with neuronal membranes is regulated by phosphorylation. Neurobiol. Aging 33, 431.e427–431.e38. 10.1016/j.neurobiolaging.2011.01.005 21388709

[B52] Poorkaj P. Bird T. D. Wijsman E. Nemens E. Garruto R. M. Anderson L.. (1998). Tau is a candidate gene for chromosome 17 frontotemporal dementia. Ann. Neurol. 43, 815–825. 10.1002/ana.410430617 9629852

[B53] Ryoo S. R. Jeong H. K. Radnaabazar C. Yoo J. J. Cho H. J. Lee H. W.. (2007). DYRK1A-mediated hyperphosphorylation of Tau A functional link between Down syndrome and Alzheimer disease. J. Biol. Chem. 282, 34850–34857. 10.1074/jbc.M707358200 17906291

[B54] Sakaue F. Saito T. Sato Y. Asada A. Ishiguro K. Hasegawa M. (2005). Phosphorylation of FTDP-17 mutant tau by cyclin-dependent kinase 5 complexed with p35, p25, or p39. J. Biol. Chem. 280, 31522–31529. 10.1074/jbc.M504792200 15994305

[B55] Sanders D. W. Kaufman S. K. DeVos S. L. Sharma A. M. Mirbaha H. Li A.. (2014). Distinct tau prion strains propagate in cells and mice and define different tauopathies. Neuron 82, 1271–1288. 10.1016/j.neuron.2014.04.047 24857020 PMC4171396

[B56] Shahpasand K. Uemura I. Saito T. Asano T. Hata K. Shibata K.. (2012). Regulation of mitochondrial transport and inter-microtubule spacing by tau phosphorylation at the sites hyperphosphorylated in Alzheimer's disease. J. Neurosci. 32, 2430–2441. 10.1523/JNEUROSCI.5927-11.2012 22396417 PMC6621814

[B57] Shukla V. Seo J. Binukumar B. Amin N. D. Reddy P. Grant P.. (2017). TFP5, a peptide inhibitor of aberrant and hyperactive Cdk5/p25, attenuates pathological phenotypes and restores synaptic function in CK-p25Tg mice. J. Alzheimers Dis. 56, 335–349. 10.3233/JAD-160916 28085018 PMC10020940

[B58] Sironi J. J. Yen S.-H. Gondal J. A. Wu Q. Grundke-Iqbal I. Iqbal K. (1998). Ser-262 in human recombinant tau protein is a markedly more favorable site for phosphorylation by CaMKII than PKA or PhK. FEBS Lett. 436, 471–475. 10.1016/S0014-5793(98)01185-5 9801171

[B59] Spillantini M. G. Goedert M. (2013). Tau pathology and neurodegeneration. Lancet Neurol. 12, 609–622. 10.1016/S1474-4422(13)70090-5 23684085

[B60] Spillantini M. G. Murrell J. R. Goedert M. Farlow M. R. Klug A. Ghetti B. (1998). Mutation in the tau gene in familial multiple system tauopathy with presenile dementia. Proc. Natl. Acad. Sci. U.S.A. 95, 7737–7741. 10.1073/pnas.95.13.7737 9636220 PMC22742

[B61] Sultan A. Nesslany F. Violet M. Bégard S. Loyens A. Talahari S.. (2011). Nuclear tau, a key player in neuronal DNA protection. J. Biol. Chem. 286, 4566–4575. 10.1074/jbc.M110.199976 21131359 PMC3039398

[B62] Tanabe K. Yamazaki H. Inaguma Y. Asada A. Kimura T. Takahashi J.. (2014). Phosphorylation of drebrin by cyclin-dependent kinase 5 and its role in neuronal migration. PLoS ONE 9:e92291. 10.1371/journal.pone.0092291 24637538 PMC3956921

[B63] Vega I. E. Cui L. Propst J. A. Hutton M. L. Lee G. Yen S.-H. (2005). Increase in tau tyrosine phosphorylation correlates with the formation of tau aggregates. Mol. Brain Res. 138, 135–144. 10.1016/j.molbrainres.2005.04.015 15913839 PMC3677942

[B64] Wada Y. Ishiguro K. Itoh T. J. Uchida T. Hotani H. Saito T.. (1998). Microtubule-stimulated phosphorylation of tau at Ser202 and Thr205 by cdk5 decreases its microtubule nucleation activity. J. Biochem. 124, 738–746. 10.1093/oxfordjournals.jbchem.a022174 9756618

[B65] Wang J. Z. Xia Y. Y. Grundke-Iqbal I. Iqbal K. (2013). Abnormal hyperphosphorylation of tau: sites, regulation, and molecular mechanism of neurofibrillary degeneration. J. Alzheimers Dis. 33, S123–S139. 10.3233/JAD-2012-129031 22710920

[B66] Wang Y. Mandelkow E. (2016). Tau in physiology and pathology. Nat. Rev. Neurosci. 17, 5–21. 10.1038/nrn.2015.1 26631930

[B67] Watanabe A. Hasegawa M. Suzuki M. Takio K. Morishima-Kawashima M. Titani K.. (1993). *In vivo* phosphorylation sites in fetal and adult rat tau. J. Biol. Chem. 268, 25712–25717. 8245007

[B68] Zempel H. Thies E. Mandelkow E. Mandelkow E. M. (2010). Aβ oligomers cause localized Ca^2+^ elevation, missorting of endogenous Tau into dendrites, Tau phosphorylation, and destruction of microtubules and spines. J. Neurosci. 30, 11938–11950. 10.1523/JNEUROSCI.2357-10.2010 20826658 PMC6633549

[B69] Zheng-Fischhöfer Q. Biernat J. Mandelkow E. M. Illenberger S. Godemann R.. (1998). Sequential phosphorylation of Tau by glycogen synthase kinase-3β and protein kinase A at Thr212 and Ser214 generates the Alzheimer-specific epitope of antibody AT100 and requires a paired-helical-filament-like conformation. FEBS J. 252, 542–552. 9546672 10.1046/j.1432-1327.1998.2520542.x

[B70] Zhou X. Z. Kops O. Werner A. Lu P. J. Shen M. Stoller G.. (2000). Pin1-dependent prolyl isomerization regulates dephosphorylation of Cdc25C and tau proteins. Mol. Cell 6, 873–883. 10.1016/S1097-2765(05)00083-3 11090625

